# Coral reef aorta: a rare form of obstruction of the ascending aorta in adolescent with aortopathy- case report

**DOI:** 10.1186/s13019-024-02553-w

**Published:** 2024-02-07

**Authors:** Neerod Kumar Jha, Benedict Raj Kumar, Nishant Shah, Osama Abdullah, Oraib Al Ketan, Fraser Harban, Mohammad Daud Khan

**Affiliations:** 1 Division of Paediatric Cardiac Surgery, Sheikh Khalifa Medical City, PO Box 768010, Abu Dhabi, United Arab Emirates; 2 Paediatric Cardiology, Sheikh Khalifa Medical City, Abu Dhabi, United Arab Emirates; 3 Core Technology Platforms, New York University, Abu Dhabi, United Arab Emirates; 4 Paediatric Cardiac Anaesthesiology, Sheikh Khalifa Medical City, Abu Dhabi, United Arab Emirates

**Keywords:** Aorta, Calcification, Coral, Reef, 3-D, Cardiac, Surgery, Printing, Modeling, Tortuosity

## Abstract

**Background:**

Supra aortic obstruction in children is uncommon and is seen in certain unique conditions. While intraluminal obstruction due to heavy calcification is seen in older populations, it is not described in pediatric populations. The coral reef aorta is a rare and distinct calcifying disease causing luminal obstruction of the suprarenal aorta in adults. The definition of this diagnosis relies entirely on the unique aspects and consistency of the lesions, which are rock-hard, irregular, gritty plaques with a white luminal surface resembling a coral reef. However, no such case has been described in children.

**Case presentation:**

We present an adolescent boy who presented with a heavily calcified ascending aortic lesion associated with aortopathy and hypertension, 12 years after an aortic coarctation repair. The investigations included echocardiography, magnetic resonance and computer-tomographic imaging. A 3-D model was printed in order to visualize and plan surgical steps in advance for safe placement of clamps and defining the extent of resection. In addition, it provided an idea about tissue quality, thickness, spatial relationship, and orientation in relation to surrounding structures. Successful resection and replacement of the diseased segment of the aorta were achieved on cardiopulmonary bypass support. Post-operative recovery was uneventful, and at 6-month follow-up, the patient is doing well. In this report, various aspects of such lesions have been discussed, including clinical presentations, complications, planning and conduct of a safe cardiopulmonary bypass, and precautions during surgery for a successful outcome.

**Conclusion:**

Complicated obstructive aortic lesions in children require careful assessment, appropriate advanced imaging, and the use of 3-D printing technology in order to plan and perform safe and effective surgical management. The etiology of severe calcified aorta in children may be related to metabolic factors, previous surgery, use of a homograft, or an inflammatory process. However, it has yet to be proven.

## Background

Supra aortic obstruction in children is uncommon and usually seen in certain unique conditions. The morphologic, anatomical, and functional abnormalities of the aorta in children and adolescents are known but, invariably, not proven to have a specific etiology on many occasions. However, genetic mutations, bicuspid aortic valves, coarctation of the aorta, and idiopathic aortopathy or tortuosity syndromes are associated with medial abnormalities of the ascending aorta [1–4]. In addition, abnormalities in the ascending aorta are prevalent in other types of patients with a variety of conditions, such as single ventricle, hypoplastic left heart syndrome, and tetralogy of Fallot. Usually, a dilatation, aneurysm, obstruction, or complication of the lesion is the reason of medical attention. There is a progressive dilatation of the aortic segment, presumably associated with reduced elasticity or increased aortic wall stiffness. The patients present with aortic dilatation, aortic aneurysm, rupture of the aorta, and calcification and may have aortic regurgitation or left ventricular hypertrophy. This new clinical entity was labeled as an aortic pathophysiological abnormality, arterial tortuosity syndrome, or “aortopathy” by many authors [4–7].

We are presenting herewith an adolescent child who presented with a calcified, obstructive ascending aortic lesion associated with aortopathy, 12-years after an aortic coarctation repair. The clinical presentation and surgical management are described with special emphasis on the role of 3-D modeling and maintenance of safe perfusion during surgery.

## Case presentation

A 12-year-old adolescent boy was lost to follow-up after a cardiac surgery done at the age of 3 months. Intraoperative findings described the presence of a thick-walled ascending aorta, napkin ring-like coarctation at the distal ascending aorta, a small aortic arch, and distinct coarctation at ductal insertion. A large pulmonary homograft patch was used to augment ascending aorta, through the aortic arch, all the way to the descending aorta. It remained unclear whether the homograft was fixed with glutaraldehyde. Recently, the patient presented with complaints of mild fatigue and occasional headache. The family history was not significant. His blood pressure was 147/93 mmHg. Auscultation revealed a 3–6 ejection systolic murmur at the left sternal border. The electrocardiogram was consistent with left ventricular hypertrophy by voltage criteria in V5 and V6. Echocardiography revealed moderate left ventricular hypertrophy, a small secundum atrial septal defect, normal Doppler interrogations across all the valves, difficult visualization of the ascending aorta, and peak instantaneous pressure gradient of 49 mmHg across it. The aortic valve was normal. There were no Marfanoid features.

**Fig. 1:**
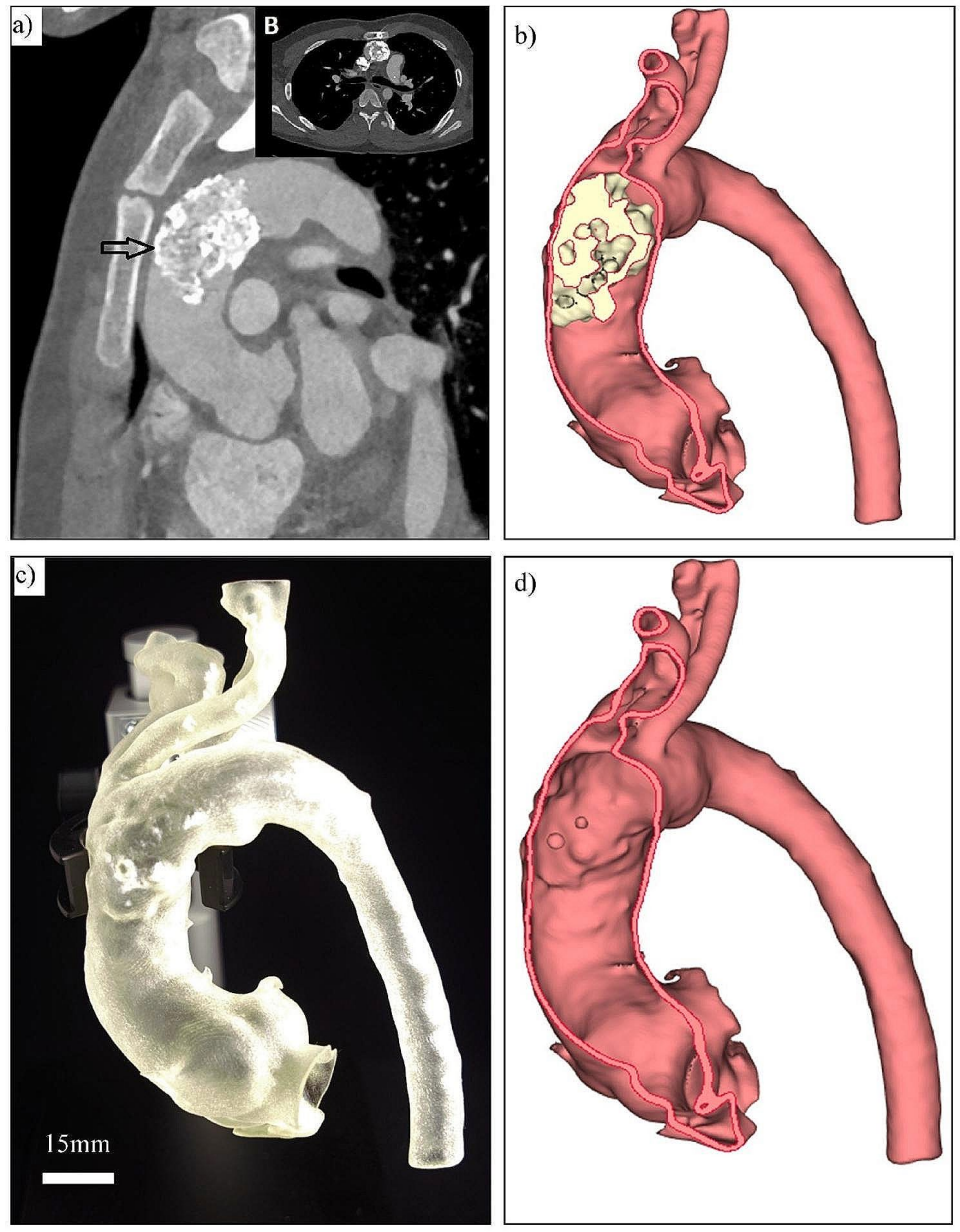
(**a**) The CT angiocardiography image showing the calcification of the ascending aorta and the arch (arrow) in sagittal and axial views (A and B). Patient-specific reconstruction of the aortic and lesion 3-D model: (**b**) segmented surface model of the aorta and the calcification region; (**c**) 3-D printed model of the aorta with vessel mimicking soft texture; and (**d**) digital surface model of the vessel aorta digital resection

**Fig. 2:**
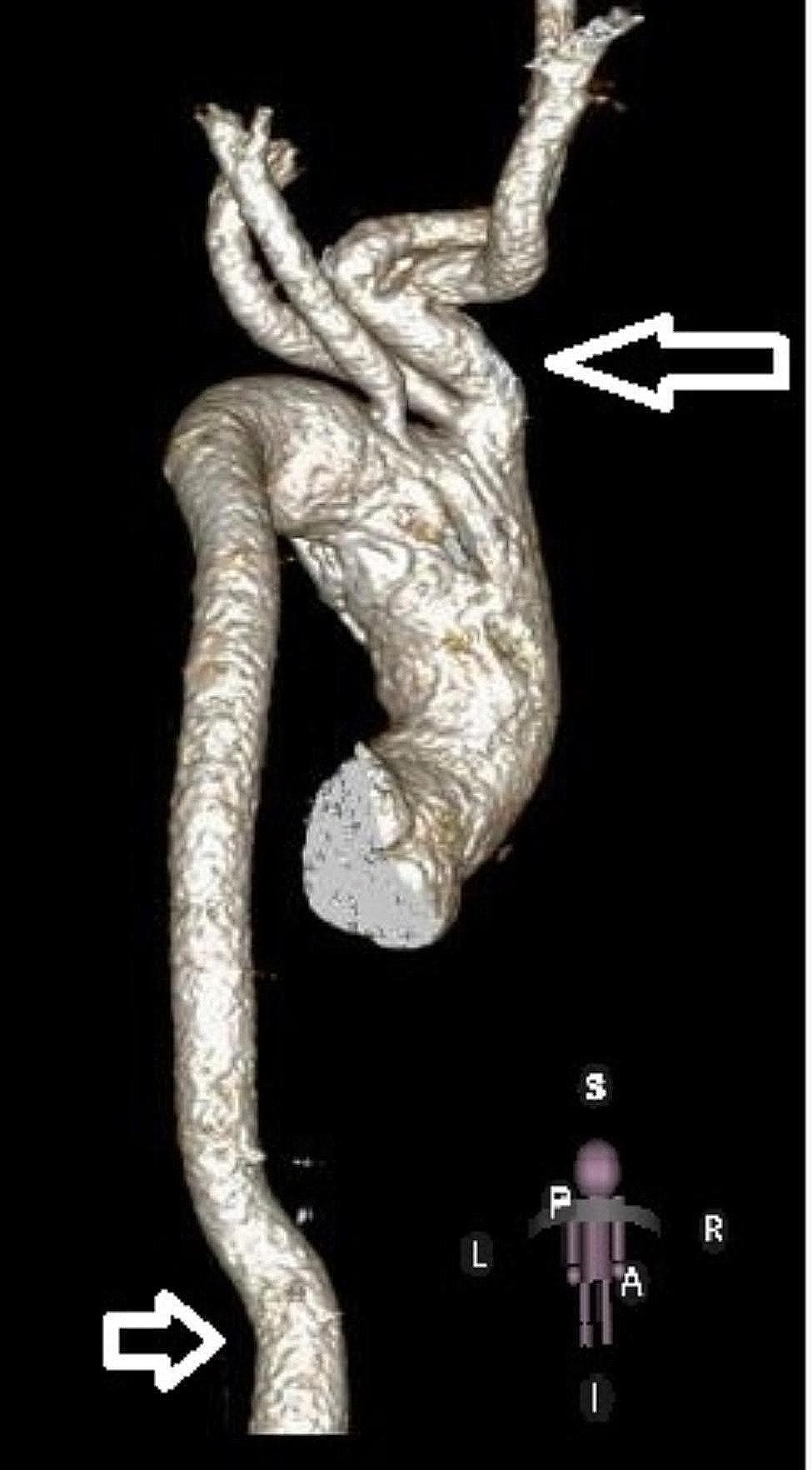
The 3-D reconstructed CT image shows a posterior view of the tortuous great vessels and the aorta (arrows). (A-anterior, P-posterior, S-superior, I-inferior, R-right, L-left)

In view of increasing gradients across the aortic arch and the inability to visualize the aortic arch due to suboptimal images, the patient warranted further investigations. Initially, cardiac magnetic resonance imaging was performed and showed irregular intra-luminal mass in the distal ascending aorta measuring approximately 38 × 24 mm, resulting in significant obstruction with blood flow turbulence. This mass was hypointense on SSFP images with a differential diagnosis of thrombus vs. calcification. Subsequently, a cardiac computerized tomography (CT) scan was performed to further delineate the extent of this mass. The CT scan including digital 3D reconstruction showed an intraluminal mass in the distal ascending aorta that is irregular in shape, attached to the anterior medial wall of the distal ascending aorta extending beyond the first branch from the arch, heavily calcified, and measuring 37 × 26 mm in size (Fig. 1a). There was a significant reduction in the ascending aorta lumen. Interestingly, the aortic arch, including branches and the descending aorta, was very tortuous (Fig. 2). This tortuosity of great vessels raised suspicion of arterial tortuosity syndrome.

Due to the presence of severe aortic obstruction and calcification, the decision was made to perform urgent surgery. Interestingly, the serum calcium levels and renal profile were normal. However, due to the complexity, a 3D model of the arch and aorta printed. The 3D-printed model helped in the planning of the suitable placement of the cross clamp and selection of aortic cannulation site for safe surgical correction. The 3D digital virtual model was reconstructed from CT images. The 3D slicer software was used to segment the CT volume into a surface model (Fig. 1b) and export it to a 3D STL model. The 3D model was then 3D printed using the Stratasys J750 Poly Jet 3D printer (Stratasys, Eden Prairie, MN, USA). Digital materials made of a mixture of TangoPlus (a soft rubber-like material) and VeroPureWhite (a rigid opaque material) were used to mimic the texture of the aorta and the calcification lesion, respectively. The FLX9740-DM with a shore-A value of 40 and FLX9750-DM with a shore-A value of 50 were used for the aorta and calcification, respectively (Shore value-hardness scale for materials 0-100, FLX-tensile and elongation strength of 3-D model). An example of the printed model is shown in Fig. 1c. To aid the surgery, a “digital resection” was planned, as shown in Fig. 1d.

**Fig. 3:**
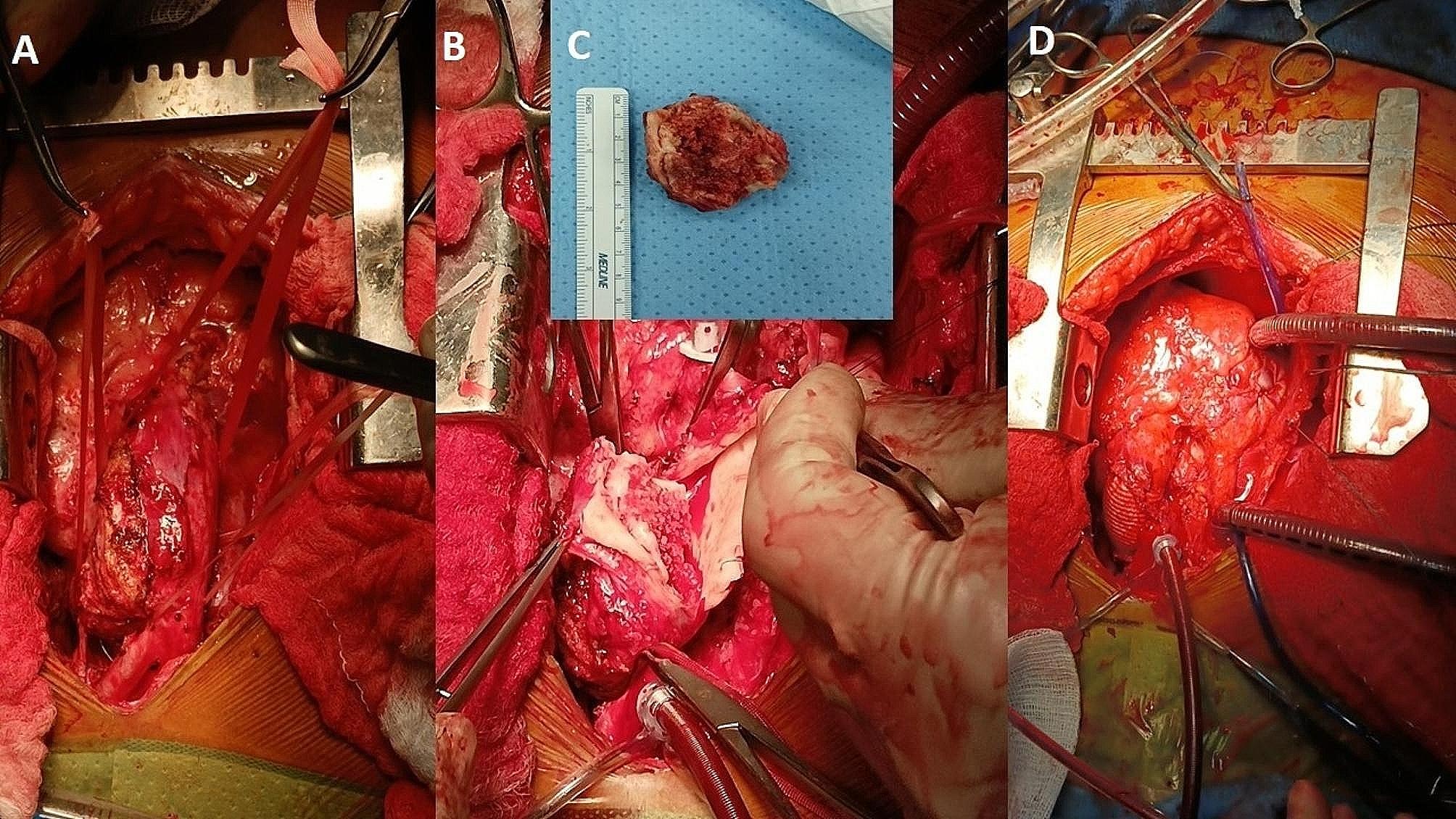
Operative photographs showing an external view of the calcified abnormal aorta (**A**), coral calcification of the lesion (**B**), an excised specimen of the calcified mass (**C**), and the position of the prosthetic graft (**D**) after reconstruction of the aorta

During surgery, a re-do sternotomy was performed. There were extensive adhesions between the heart and the surrounding tissue in the lungs that were dissected successfully. The calcified ascending aorta was severely adhered to the upper part of the sternum. The innominate artery was cannulated for antegrade perfusion. In order to perfuse the distal aorta, we also had to cannulate the left femoral artery, and then both were connected to the cardiopulmonary bypass circuit under systemic heparinization. In fact, it is an example of an extra-anatomical bypass. The elongation, tortuosity, and clustering of great artery origins were evident on inspection. The superior vena cava and inferior vena cava were also cannulated. After establishing full cardiopulmonary bypass, the three areas were cross-camped. First, proximal to the diseased area on the ascending aorta distal to the cardioplegia delivery site. The second clamp was placed at the origin of great vessels (the bovine trunk), and the third clamp was placed distal to the calcified segment on the proximal descending aorta. Subsequently, the custodial cardioplegia was delivered through the aortic root. The placement of the aortic cross clamps was based on various factors, such as findings from inspection, palpation, and information obtained during 3-D modeling and CT angiography. The aim was to maintain perfusion of the head, neck, and lower body, avoid injury to the diseased area, obtain a bloodless field for surgery, and avoid embolism. The right atriotomy was then performed to close the atrial septal defect directly. The systemic hypothermia (20 °C) was achieved. We then excised the calcified segment that involved the distal ascending aorta under the surface of the arch and a little bit of the proximal descending thoracic aorta (Fig. 3). All diseased areas, including thickened wall, were excised, and irrigation with normal saline was done to prevent any embolism. Finally, a woven double velour vascular graft (24 mm Hemashield Platinum, M/s Intervascular, France) was used to perform end-to-end anastomosis to replace the abnormal aortic segment (Fig. 3). The de-airing was performed, and the patient could be weaned from the cardiopulmonary bypass, after rewarming. The tissue histopathology of excised segment confirmed calcification, necrosis and medial thickening with fibrotic changes.

The postoperative period was uneventful, and recovery was satisfactory. The patient was discharged home two weeks after surgery on antihypertensive medications. The echocardiography at discharge and 6-months later revealed a satisfactory repair with no residual lesions.

## Discussion

In the coral reef aorta, heavily calcified plaques grow into the lumen more extensively than those observed in routine atherosclerosis, and the lesions tend to be located on the posterior surface of the aorta. In our case, we speculate that extensive calcification growth might have occurred in the homograft material used during repair of the coarctation of the aorta during infancy.

The integrity of the vascular structures is dependent on multiple factors that include connective tissue elements, cardiovascular abnormalities, genetic anomalies, and acquired elements such as trauma, surgical interventions, and the use of prosthetic materials [4–7]. Calcification of a valve conduit homograft is a very well-known complication, particularly when used in the aortic place [8]; however, the extent of calcification is limited and does not cause significant hemodynamic instability. An intraluminal calcification related to homograft patch leading to severe obstruction has not been reported, yet.

Although there is some debate about inflammation and homograft dysfunction, numerous reports implicate inflammatory processes in bioprosthetic valve failure [9]. We believe that inflammatory processes in the past may have been partly responsible for the pathology seen in this case, owing to the severe hyalinization, rapid growth, luminal projections, and severe extensive calcification of the homograft.

Arterial tortuosity syndrome is a rare hereditary autosomal recessive connective tissue disorder characterized by unusual tortuosity, elongation, distortion, and folding of major arteries, resulting in vascular dilatation, stenosis, and the formation of aneurysms. Large and medium-sized arteries are mostly affected. In the majority of the patients, there is cardiomegaly with ventricular hypertrophy (10–11). Our patient had evidence of aortic and great vessel’s tortuosity, as evident on the CT scan. The operative findings during the first and redo surgeries also confirmed the presence of vascular tortuosity and thick vessel walls. Clinically suspected arterial tortuosity syndrome may have played a role in the etiopathogenesis. Unfortunately, genetic testing for arterial tortuosity syndrome could not be performed due to financial coverage. In our opinion, the recurrence of vascular dilatation and distortion at the previous surgical site and subsequent changes such as calcification, thickening and obstruction may be correlated with abnormal connective tissues within the vasculature.

The risk of re-doing sternotomy in the presence of adhesions, aneurysmal aortic dilatation, abnormal vasculature, and calcification is profound in terms of complications such as bleeding, rupture, or embolization. In this context, a preoperative workup should not only include an extensive evaluation of the anatomy by imaging, but 3D modeling and printing technology is invaluable to identify critical elements to address during surgery such as the safest site for placement of occlusive clamps, selection of perfusion strategy or tissue handling for a safe surgery and better outcome. The 3D printing technology also offers the possibility to virtually explore the intra-cardiac structures and their relationships, as well as the size and course of blood vessels, including the coronary arteries. Additionally, calcification, nature of the tissues and difficult areas can be identified [12].

Long-standing hypertension is a well-known complication of aortic diseases in adulthood, including aortic coarctation or aneurysm. It could be attributed to the effects of mechanical obstruction, changes in the aortic wall, altered baroreceptor mechanisms, and physiological mechanisms involving renal and endocrine systems [13]. However, hypertension can be a consequence of aortic disease or vice versa.

## Conclusion

Complicated obstructive aortic lesions in children require careful assessment, appropriate imaging, and selective use of the 3-D printing technology for effective planning and a safe surgical management. The etiology of severe calcified aorta in children may be related to aortopathy, metabolic factors, previous surgery, use of a homograft, or an inflammatory process. However, it has yet to be proven.

## Data Availability

Not applicable.

## Abbreviations

3D: Three-dimensional
CT: Computer tomography
